# Advanced Sinonasal Malignant Melanoma; A Case Report

**DOI:** 10.30699/IJP.2023.553175.2987

**Published:** 2023-07-16

**Authors:** Parisa Khorasani Esmaili, Shahriar Dabiri, Ayeh Shamsaddini, Touraj Reza Mirshekari

**Affiliations:** 1 *Department of Pathology, Pathology and Stem Cells Research Center, Afzali Pour Medical Faculty, Kerman University of Medical Sciences, Kerman, Iran*; 2 *Department of Otorhinolaryngology, Shafa Hospital, Kerman University of Medical Sciences, Kerman, Iran*; 3 *Department of Pathology, Shafa Hospital, Kerman University of Medical Sciences, Kerman, Iran*

**Keywords:** Epistaxis, Malignant melanoma, Mucosal melanoma, Sinonasal

## Abstract

Malignant melanoma of the sinonasal area is a rare tumor that arises from melanocytes in the nasal mucosa and is more aggressive than the cutaneous type with a poor prognosis.

We report a 60-year-old female with the initial chief complaint of nasal cavity fullness, continuous epistaxis, and nasal bone deformity in the past two months.

In a primary examination, a black mass was found, and in an excisional biopsy, the pathologist reported sinonasal malignant melanoma, which was confirmed after IHC staining.

In spindle cell tumors of the head and neck area, we should be aware of mucosal malignant melanoma as a differential diagnosis.

## Introduction

Malignant Melanoma of the nasal cavity as a primary tumor is very rare and has more aggressive behavior and more poor prognosis than its cutaneous form. Primary malignant melanoma of the nasal cavity arises from melanocytes located in the mucous membrane. Only 0.5% of malignant melanoma arises in the nasal cavity and it should be in the differential diagnosis of nasal and paranasal sinus tumors (1).

Usually, it presents in advanced stages with a fatal course. In the amelanotic variety, diagnosis becomes more difficult and may need immunohistochemistry (IHC) staining. Radical surgery increases morbidity; moreover, radiotherapy and chemotherapy have little role (2).

Local recurrence and metastasis are common and tumor resection with free surgical margins is the most important prognostic factor in the nasal cavity. early diagnosis, radical surgery, and radiotherapy can play effective roles, and cell and gene therapy are currently under evaluation in this tumor (3).

## Case Presentation

We report A 60 years old woman with a nasal cavity mass and primary presentation of nasal cavity fullness, continuous epistaxis, and nasal bone deformity in the recent 2 months who was referred to the otorhinolaryngology clinic of Shafa Hospital (Kerman-Iran).

In past medical history, she mentioned only increased hypertension (under treatment with losartan tab.25mg) without a history of previous malignancy, especially cutaneous melanoma.

**Fig. 1:**
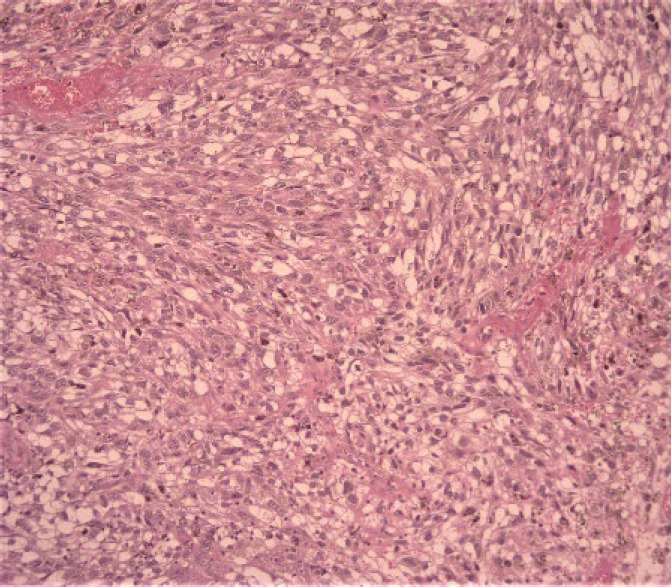
A spindle neoplastic growth with foci of epitheloid feature and prominent nuclear pleomorphism (x10 magnification)**.**

**Fig. 2:**
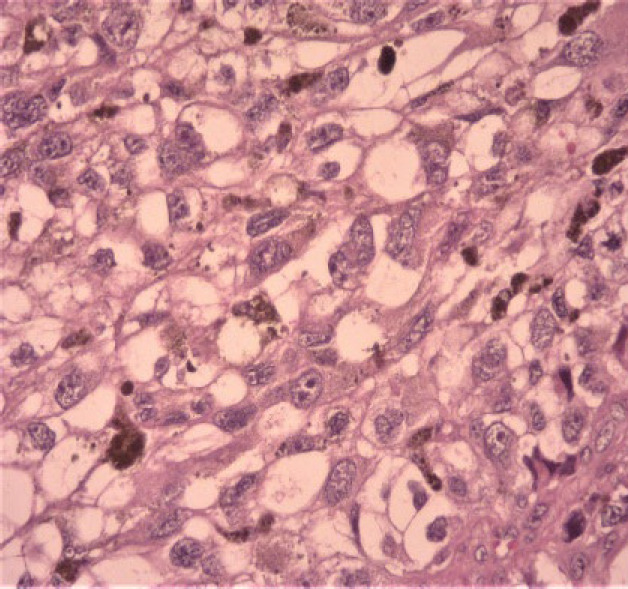
Melanin depositions and atypia (x40 magnification).

**Fig. 3:**
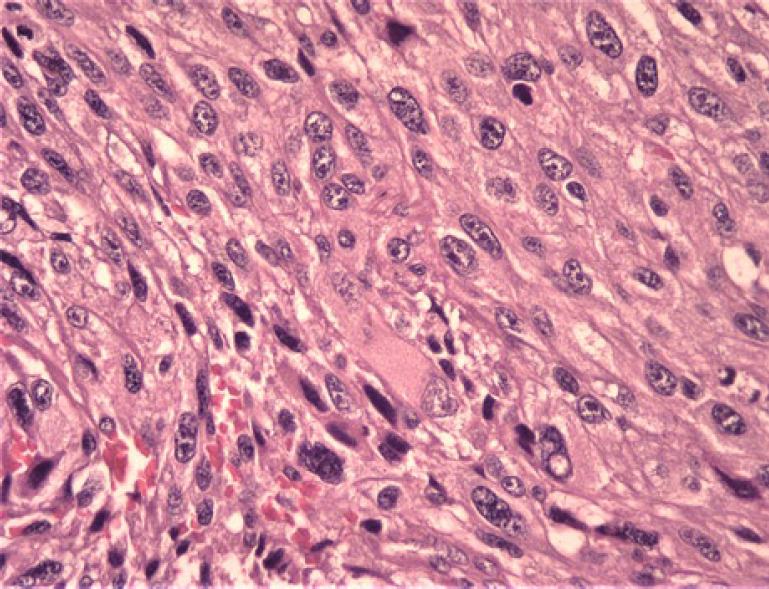
Mitosis in tumor cells is prominent (x40 magnification).

In the physical examination of the skin, there was no lesion or a suspicious primary tumor cell. Only nasal bone deformity was obvious and in sinus endoscopy, a black-brown mass in nasal cavity-derived nasal septa and other physical examinations was normal. In an excisional biopsy, a neoplastic growth with a whorling pattern composed of malignant nevoid cells with mixed features containing spindle and epithelioid shapes, marked melanin deposition, prominent red nucleoli, and nuclear pleomorphism that suggest malignant melanoma as one of differential diagnosis, was found (Figures 1, 2, and 3).

In histopathological evaluation, we found an ulcerative neoplastic growth that started from the mucosal epithelial surface and grew downward. atypical mitosis was seen and the mean mitotic count was 2 mitosis/HPF.

**Fig. 4:**
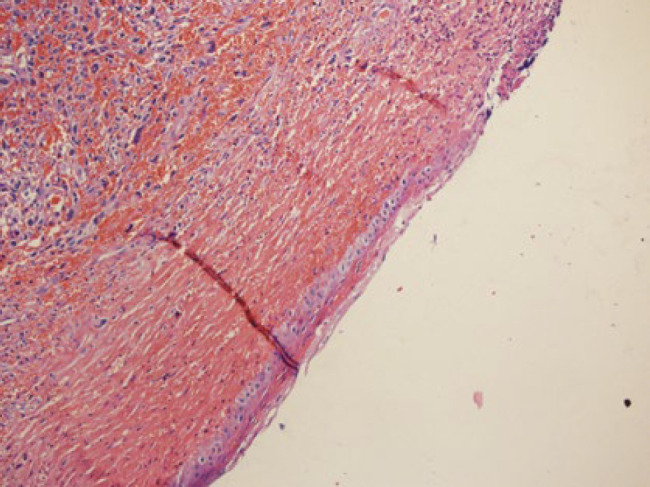
Spindle malignant cells with mucosal epithelial ulceration, surface involvement, and marked congestion (x10 )

**Fig. 5:**
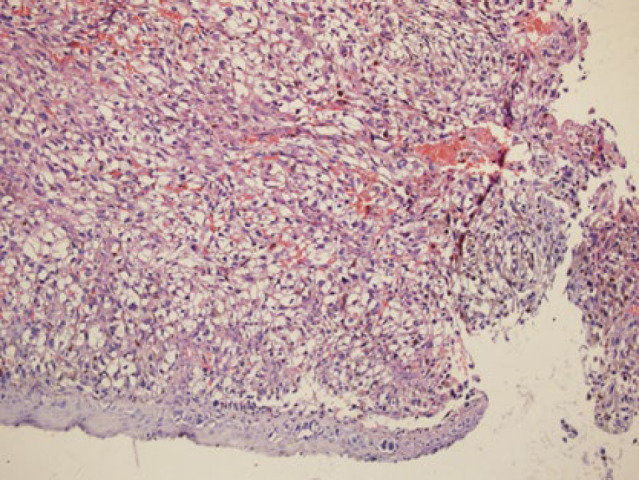
Spindle malignant cells with mucosal epithelial ulceration, surface involvement, and marked congestion (x10 )

**Fig. 6:**
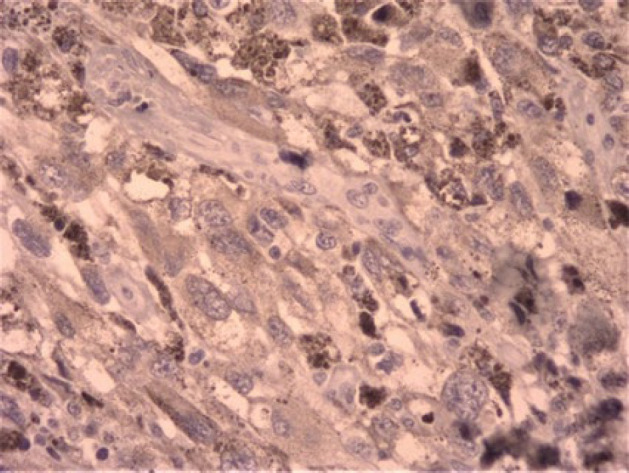
The positive reaction of tumoral cells for HMB-45 also melanin deposition is remarkable (x40 magnification).

**Fig. 7:**
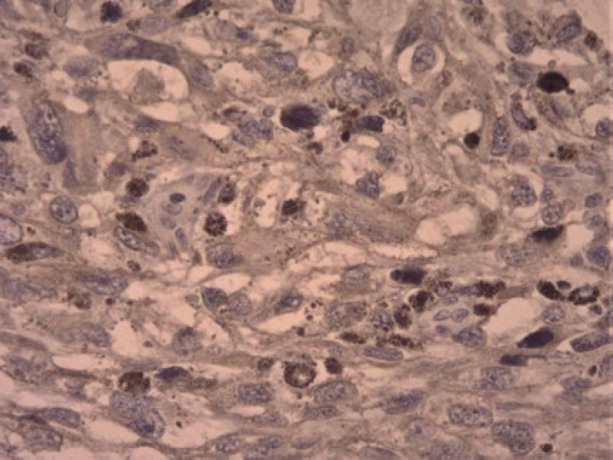
The positive reaction of tumoral cells for HMB-45 also melanin deposition is remarkable (x40 magnification).

**Fig. 8:**
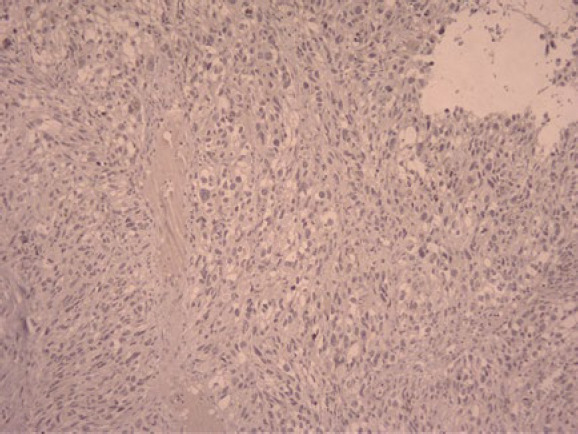
IHC for CK shows a negative reaction in tumor cells

**Fig. 9:**
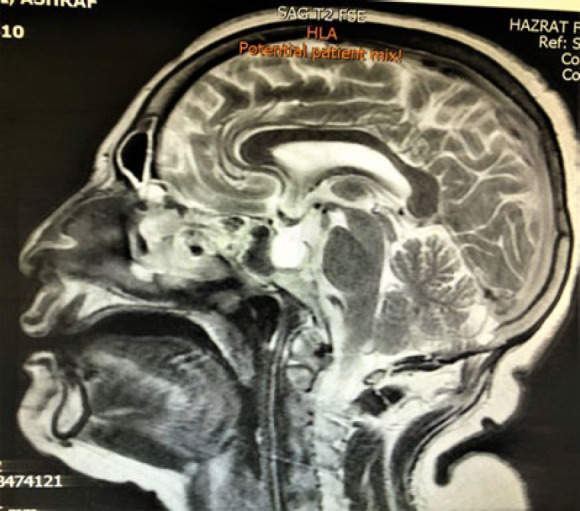
MRI imaging shows the primary mass in the nasal cavity, extended to the nasal bone with foci of metastasis in the brain

**Fig. 10:**
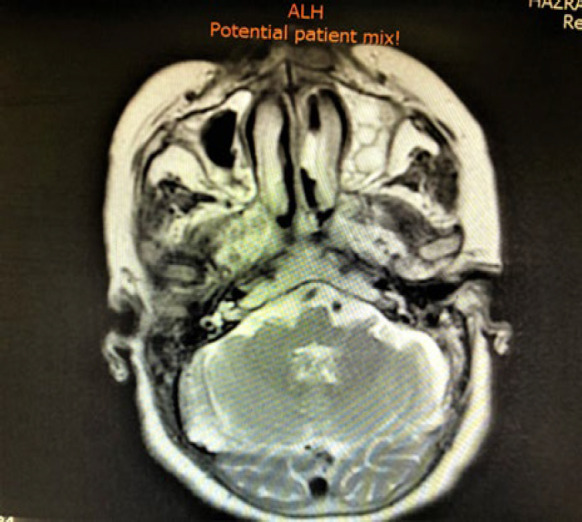
MRI imaging shows the primary mass in the nasal cavity, extended to the nasal bone with foci of metastasis in the brain

IHC results in Figures 6 and 7 show positive staining for HMB-45 (Human Melanoma Black45) and negative reaction for CK(Cytokeratin) and EMA (Epithelial Membrane Antigen) in tumor cells, which confirmed our diagnosis (Figure 8).

After that evaluation for tumor extension and finding metastasis was started in the head and neck area imaging showed foci of metastatic tumor site in brain MRI and tumor extension in the nasal cavity with nasal bone destruction and invasion to the base of the skull was found (Figures 5 and 6). 

The Patient was a candidate for surgery and chemo-radiotherapy. A few months after diagnosis patient died.

## Discussion

Most of the malignancy of nasal mucosa presented with nasal obstruction, epistaxis, and back drip. Usually, at first, clinicians think about polyposis but it needs to take a sample biopsy to rule out malignancy (4).

Nasal mucosa melanoma is one of the rare malignancies in this area which presents with nasal obstruction, rhinorrhea, and epistaxis (abundant or minimal with the presence of streaks of blood when blowing the nose. These are the nonspecific symptoms and may be delayed in diagnosis because of this reason. the advanced tumor presented with nasal deformity and malar swelling or exophthalmos (3,5).

 Malignant melanomas have 2 origins: cutaneous and mucosal. The mucosal form has a worse prognosis because of its aggressiveness compared with that of the cutaneous form. Mucosal melanomas often occur at a rate of 2% to 3% among all melanomas and are typically found in the nasal cavity and paranasal sinuses (6,7).

Sinonasal melanoma seems to be incurable with a poor prognosis, First-line treatment consists of surgery. Intranasal endoscopic surgery remains controversial due to the difficulty of controlling surgical margins. Adjuvant radiotherapy performed efficacy on local and regional disease control. Five-year overall survival of mucosal melanoma of the nasal cavity and paranasal sinuses is about 40%. Local recurrence is observed in about 50% of cases and metastatic disease is common (3,8,9,).

Unlike skin melanomas, there are no risk factors and the disease is frequently manifested in older patients, (50-70 years). usually diagnosed in advanced stages and unfortunately, they are resistant to chemo and radiotherapy .. Immunotherapy has not been useful in the treatment of mucosal melanomas (10,11).

The most common sites of mucosal melanomas in the upper airways are the oral cavity, the nasal cavity, and the paranasal sinuses. Most of the cases affect the nasal septum, the inferior conchae, and the middle conchae (11). 

In histopathology examination, it can be similar to undifferentiated carcinoma, but immunochemical analysis revealed positive immunostaining for HMB-45, Melan-A, S-100, Vimentin, cyclin D1 and CD44 markers in tumor cells (12,13).

prognostic factors include endoscopic or endoscopic‑assisted surgery were the preferred methods of treatment, and histological features, including the presence of tumor melanin pigmentation and pathologic staging (14,15).

## Conclusion

Malignant melanoma of the nasal cavity is a rare aggressive tumor and also presents with nonspecific symptoms, we should be aware that early diagnosis of tumor in this region is very important. Also In spindle cell tumors of the head and neck area, we should consider mucosal malignant melanoma.

## Funding

 None.

## Conflict of Interest

There are no conflicts of interest.
